# Supplementary figures and images for: Reducing malaria burden and accelerating elimination with long-lasting systemic insecticides: a modelling study of three potential use cases
Source: Malar J. 2019 Sep 5;18:307. doi: 10.1186/s12936-019-2942-4 (PMC6727392; doi:10.1186/s12936-019-2942-4)

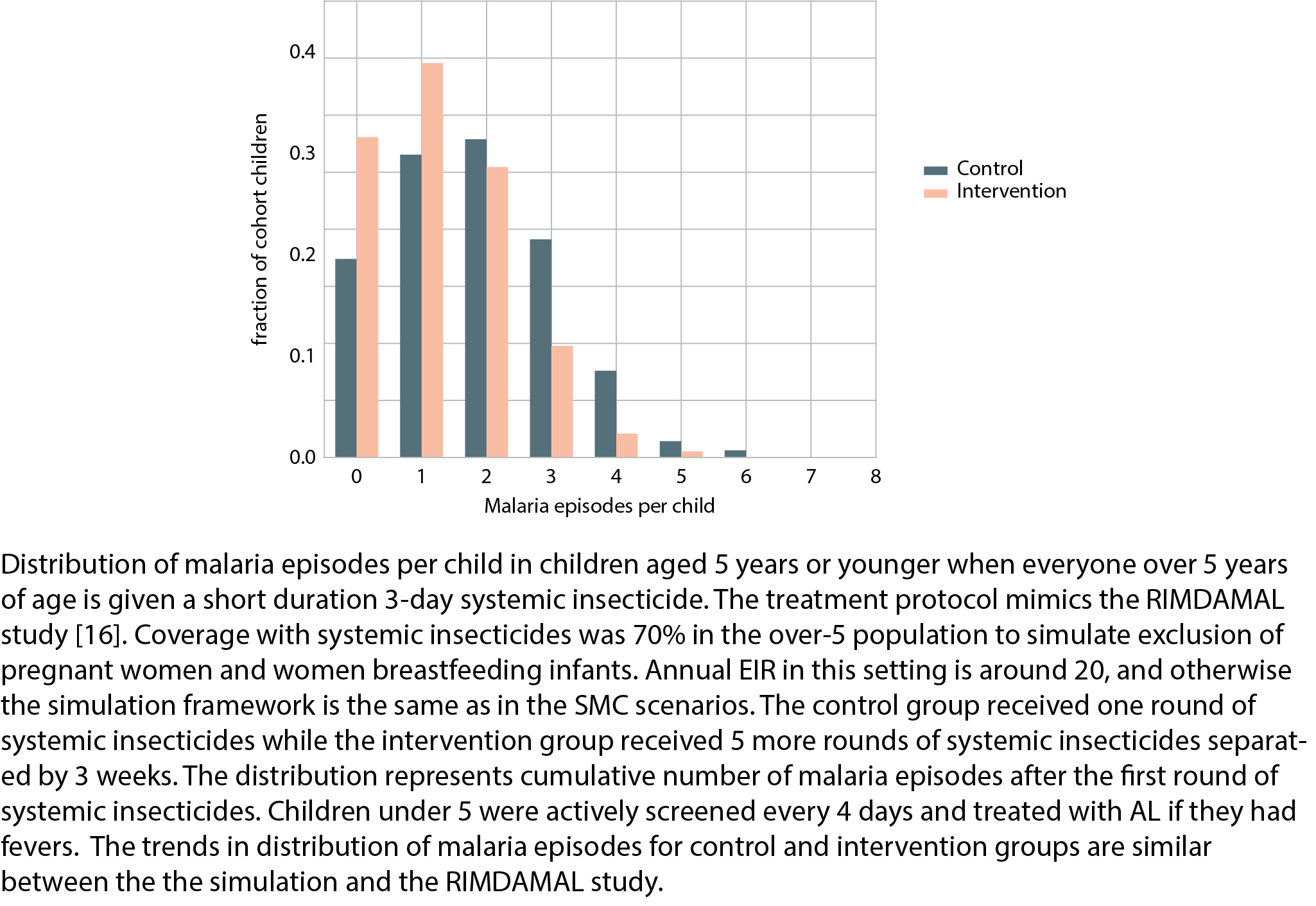

Supplement: Supplementary file 1 — Additional file 1. Distribution of malaria episodes per child in a simulation mimicking the RIMDAMAL study. [file 12936_2019_2942_MOESM1_ESM.png]

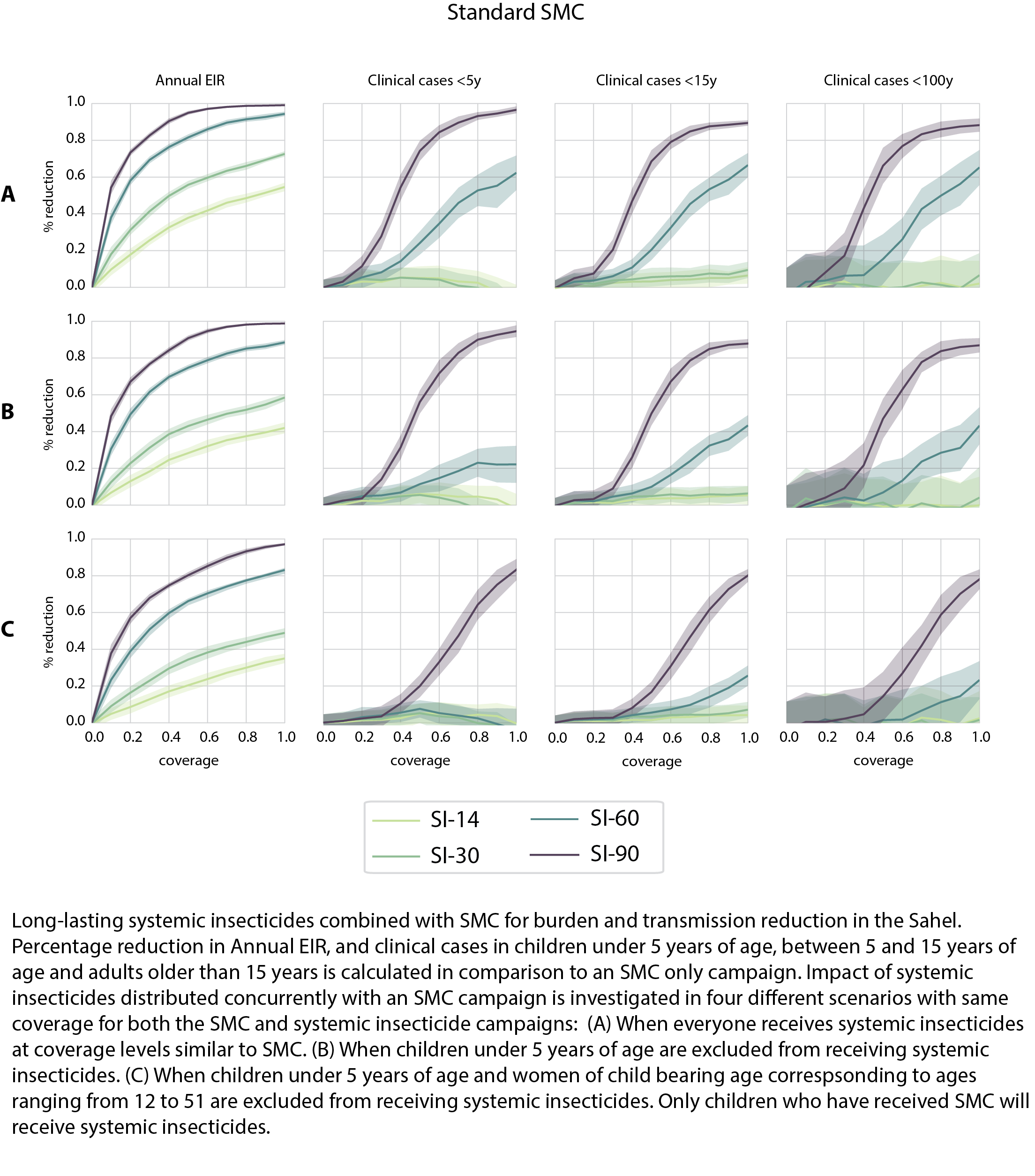

Supplement: Supplementary file 2 — Additional file 2. Impact of long-lasting systemic insecticides combined with standard SMC on annual EIR and case reduction by age group. [file 12936_2019_2942_MOESM2_ESM.png]

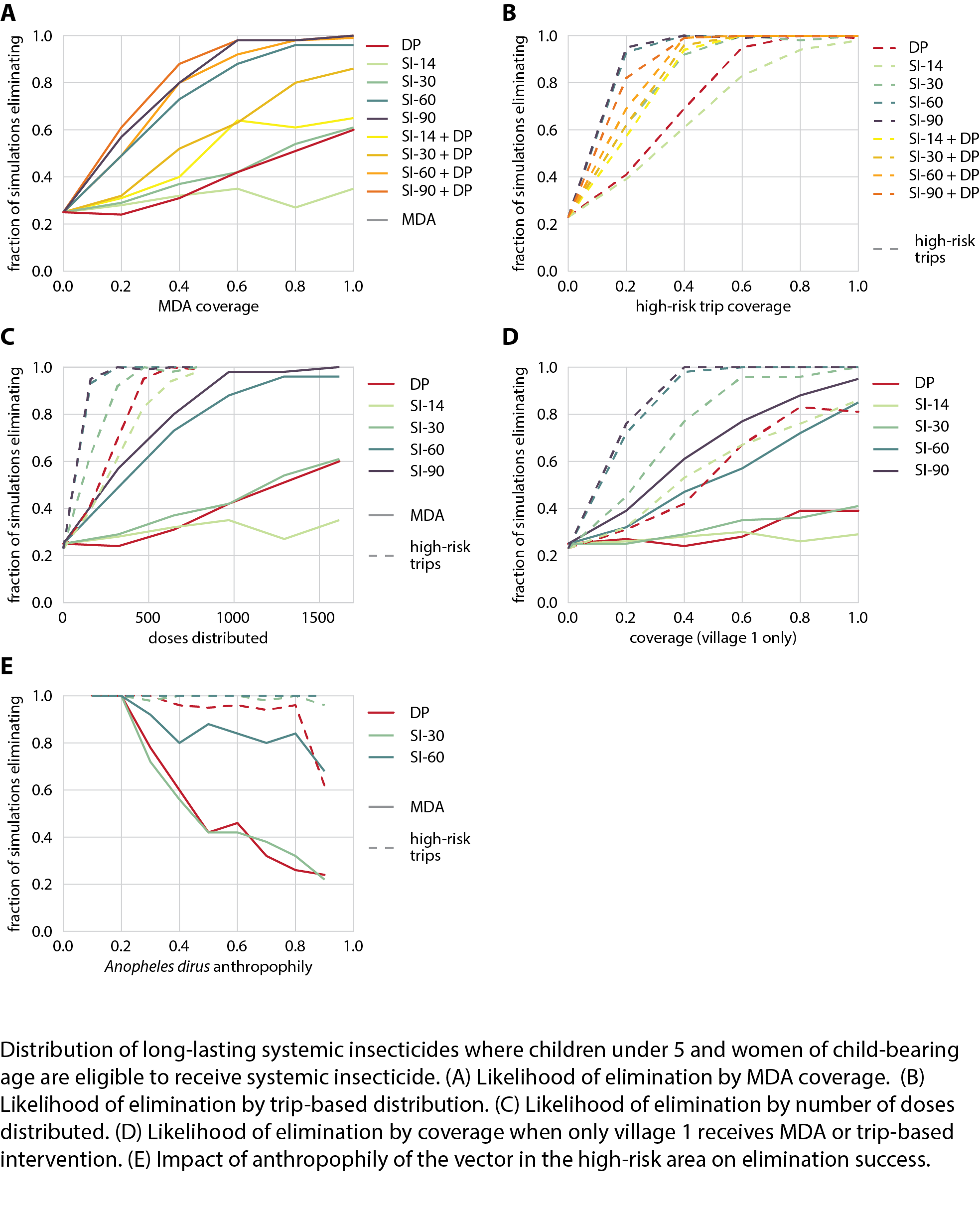

Supplement: Supplementary file 3 — Additional file 3. Impact of long-lasting systemic insecticides in a targeted elimination scenario where children under 5 and women of childbearing age are eligible to receive systemic insecticide. [file 12936_2019_2942_MOESM3_ESM.png]
